# Recognition of dynamic facial expressions of emotions in forensic inpatients who have committed sexual offenses: a signal detection analysis

**DOI:** 10.3389/fpsyt.2024.1384789

**Published:** 2024-06-13

**Authors:** Luca A. Tiberi, Steven M. Gillespie, Xavier Saloppé, Audrey Vicenzutto, Thierry H. Pham

**Affiliations:** ^1^ Forensic Psychology Department, University of Mons (UMONS), Mons, Belgium; ^2^ Department of Primary Care and Mental Health, University of Liverpool, Liverpool, United Kingdom; ^3^ Center of Research in Social Defense, Tournai, Belgium; ^4^ Univ. Lille, CNRS, UMR 9193 - SCALab - Sciences Cognitives et Sciences Affectives, Lille, France; ^5^ Psychiatric Unit, Saint-Amand-les-Eaux Hospital, Saint-Amand-les-Eaux, France

**Keywords:** Emotions, facial expressions of emotion, sexual offending, Forensic inpatient, Signal detection theory

## Abstract

Emotion recognition is central in prosocial interaction, enabling the inference of mental and affective states. Individuals who have committed sexual offenses are known to exhibit socio-affective deficits, one of the four dynamic risk assessment dimensions found in the literature. Few research focused on emotion recognition. The available literature, exclusively on individuals in prison who have committed sexual offenses, showed contrasting results. Some found a global (across all emotions) or specific (e.g., anger, fear) deficit in emotion recognition. In contrast, others found no difference between individuals in prison who have committed sexual offenses and those who have committed non-sexual offenses. In addition, no such study has been undertaken among forensic inpatients who exhibit socio-affective deficits. This study aims to investigate the recognition of dynamic facial expressions of emotion in 112 male participants divided into three groups: forensic inpatients who have committed sexual offenses (*n* = 37), forensic inpatients who have committed non-sexual offenses (*n* = 25), and community members (*n* = 50), using the Signal Detection Theory indices: sensitivity (*d*’) and response bias (*c*). In addition, measures related to reaction time, emotion labeling reflection time, task easiness, and easiness reflection time were also collected. Non-parametric analyses (Kruskall-Wallis’ *H*, followed by Mann-Whitney’s *U* with Dunn-Bonferroni correction) highlighted that the two forensic inpatient groups exhibited emotion recognition deficits when compared to community members. Forensic inpatients who have committed sexual offenses were more conservative in selecting the surprise label than community members. They also took significantly more time to react to stimuli and to select an emotional label. Despite emotion recognition deficits, the two forensic inpatient groups reported more stimuli easiness than community members.

## 1 Introduction

Sexual offending is a complex, multifaceted phenomenon that captures a variety of contact and non-contact sexually oriented behaviors perpetrated by individuals with different sets of motivations, attitudes, and beliefs (1, 2). The term *sexual offenders* is typically used to refer to individuals *who have been charged or committed a sexual offense*. However, guidelines from the *Association for the Treatment and Prevention of Sexual Abuse* promote using person-first language in accordance with ethical principles in forensic psychological research and practice (3). In accordance with these guidelines, we will use the terms *individuals who have committed sexual offenses* or *individuals who have committed non-sexual offenses*. The problem of sexual offending is a considerable societal, economic, and public health concern, and research has focused on identifying those factors that can help to predict the risk that an individual who has committed a sexual offense may do so again in the future.

One framework for understanding these factors, or criminogenic needs, is the *Structured Risk Assessment*, later renamed *Structured Assessment of Risk and Need* (SARN) (4). This risk assessment framework outlines four domains of ‘dynamic’ risk factors (i.e., those risk factors that are changeable and can be modified in treatment) that are known to predict recidivism risk: sexual interests, distorted attitudes, self-management, and socio-affective functioning. While each domain has received attention in the literature, the role of emotion in sexual offending is not well understood.

Socio-affective functioning refers to the ways in which people interact with others, and the emotional and motivational processes underlying these interactions (4). One aspect of socio-affective functioning that has received some attention is emotion recognition. Emotion recognition is the competency to accurately perceive, discriminate, categorize, and label an emotion (5), and is central to appropriate human social interactions. The competency of an observer to recognize another’s emotional expressions (whether expressed via facial expressions, audibly, or in bodily postures) enables the observer to label and understand the sender’s emotional state and to regulate their own mental and affective states in response to the other’s emotions (6). The *Tripartite Emotion and Expression Perception* model offers a theoretical framework for understanding emotion recognition, encompassing the three major communication channels: face, voice, and body posture (7). The ‘Tripartite’ notion refers to three functions of an expressed emotion, which (i) serves as a *symptom* of an emotional event from the sender, (ii) *appeals* to the attention of the observer, and (iii) *symbolizes* a meaning (8). Emotion recognition is therefore conceptualized as a three-step process: a) the production of distal cues, such as face, voice, and body posture expression; b) transmission, including depletion of cue quality (or ‘noise’); and c) proximal perception through visual or auditory channels (7, 8). The final step, perception, allows for recognition of the expressed emotion through appraisal, or the cognitive evaluation of a stimulus or event (9).

Several theories have attempted to explain how problems in the processing and recognition of others’ emotional facial expressions may be associated with behaviors that threaten or cause harm to others. For example, in one prominent theory of psychopathic personality, it is suggested that psychopath’s impaired recognition of others’ fearful and sad facial expressions of emotion may be associated with problems recognizing and understanding another’s distress e.g., in response to threats or violence (10, 11). This can lead to a failure of the violence inhibition mechanism, whereby one usually learns to experience others’ distress as aversive and, through a process of socialization, learns to avoid behaving in threatening or aggressive ways that cause distress or sadness. It has been suggested that similar impairments in recognizing others’ facial expressions of emotion may characterize people with a history of sexual violence and represent a dynamic risk factor for sexual offense recidivism, consistent with problems in socio-affective functioning in this population (12).

Research on emotion recognition competency among individuals who have committed sexual offenses has mainly focused on the recognition of facial expressions of emotion in prison samples and has revealed conflicting results (13, 14). Most of these studies have reported lower accuracy and sensitivity to emotional expressions among individuals who have committed sexual offenses compared to individuals who have committed non-sexual offenses (12, 15–18) or community members (12, 15–17, 19–21). Problems recognizing others’ emotional expressions among individuals who have committed sexual offenses have been found on a global level (across various emotional expressions), with problems reported for fear (12, 18–21), disgust (12, 19–21), anger (12, 15, 18), and surprise (15, 18). In contrast, other studies have found generally preserved or even heightened competencies for emotional expression recognition (22–24).

Despite a growing body of research, considerable variability remains in (a) experimental and control populations identified for recruitment, (b) materials/stimuli used, and (c) methods of analysis. At first glance, this leads to difficulties drawing comparisons across studies. First, several studies gathered individuals who have committed sexual and non-sexual offenses in the same group (16, 18–20), increasing participant profile variability. The compositions of these samples preclude any solid conclusions about the specific emotion recognition competencies of individuals who have committed sexual offenses. Other differences in sample composition relate to inclusion decisions based on victim type, with some studies failing to differentiate between those with adult victims compared to child victims (12, 24), and those with/out a diagnosis of pedophilia or other paraphilia (21). Again, the heterogeneity of these samples may help to explain mixed patterns of results and leads to difficulty when considering implications for assessment and treatment.

Second, most of the studies that examined facial expression recognition have used static, monochromatic, Caucasian, male and female stimuli, with the most frequently used stimulus set being the *Pictures of Facial Affect* (POFA) (25). More recent studies have used static, colorized, multi-ethnic stimuli, including images from the *NimStim Set of Facial Expressions* (26), or the *Radboud Faces database* (27), with some articles reporting the use of a morphing technique to create static stimuli varying in intensity, from a neutral facial expression (0% intensity) to a prototypical facial expression (100% intensity) (12, 22). None of the studies with individuals who have committed sexual offenses have used morphing techniques to create dynamic stimuli, depicting the temporal emergence of a facial expression of emotion, starting with a neutral expression, and concluding with a prototypical emotion. Variations in sampling and stimuli may help to account for mixed and often contradictory findings in the literature. For example, the dynamic, temporal unfolding of facial expressions of emotion increases recognition accuracy despite the invariance of specific emotion thresholds for recognition (28). This effect is referred to as the *dynamic recognition advantage*. It assumes that motion aids attentional capture and focus, and enhanced attention facilitates early perceptual processing (28, 29). Dynamic facial expressions of emotion also echo the context of everyday social interactions, where emotions are rarely prototypical, are expressed with a temporal course, and vary in intensity, aiding greater ecological validity (29). Besides the temporal unfolding of the facial expression, differences in the gender of stimuli may also contribute to conflicting results. For example, Wells et al. (30) found that female faces were recognized more accurately but less rapidly compared to male faces. Other paradigmatic discrepancies relate to the response options. Some studies have offered up to six (17–21, 23) or seven (12, 15, 24) response options, inclusive of neutral and the so-called ‘discrete’ expressions of emotion (anger, disgust, fear, happy, sadness, and surprise), while others have used forced-choice paradigms with only two (anger and fear) response options (24) or matched-choice paradigm (17) (see (13) for a review).

Thirdly, a variety of outcome measures and statistical procedures have been employed. While most studies computed mean scores for proportion correct, others (12, 15, 17) employed the *Signal Detection Theory* (SDT) (31, 32) to analyze four outcomes in the decision-making process: Hit Rate (HR), when a signal is accurately recognized as a “*signal*”; False Alarm (FA), when a noise is inaccurately recognized as a “*signal*”; Correct Rejection (CR), when a noise is accurately identified as a “*noise*”; and finally, Miss (M), when a signal is inaccurately recognized as “*noise*.” HR and FA scores enable the computation of two indices: sensitivity and response bias. Sensitivity [*d’* = *z*(HR) – *z*(FA)] refers to the participant’s competency to accurately discriminate the signal (stimulus) from the noise. A low *d’* value (tending to 0) indicates poor sensitivity, while higher *d’* values (tending to +∞) indicate increasing sensitivity to distinguish between differing emotions. On the other hand, response bias (*c* = - (*z*HR + *z*FA)/2) refers to the participant’s response style. It is computed based on the difference between the subjective criterion (β) and a hypothetical criterion (β) of an unbiased observant with equal proportions of misses and false alarms (33). In other words, the participant response style is assessed as liberal if *c* is negative (tending to -∞), meaning that the participant selects the emotion label often without specificity (high HR, FA, and low M and CR). A more conservative response style will be reflected in positive values of *c*, tending to +∞, meaning that the participant rarely selects the emotion label (low HR, FA, and high M and CR). Studies using SDT mainly suggest that individuals who have committed sexual offenses show generally lower sensitivity to emotional expressions than either individuals who have committed non-sexual offenses or men from the community (15, 17), while one study highlighted that sensitivity varied depending on the emotional content and the gender of the stimulus (12). Regarding response bias, it has been shown that individuals who have committed sexual offenses are more conservative in choosing the sadness label (15), while individuals who have committed non-sexual offenses are more conservative in selecting the fear label, especially at higher intensities of emotional expressiveness (12).

Because of mixed findings in the literature, the research question in this study asked whether or not there are differences in the processing of others’ facial expressions of emotion between forensic inpatients who have committed sexual offenses, compared with forensic inpatients who have committed non-sexual offenses, and community members. For this purpose, we used dynamic emotion expression stimuli, and several indicators to assess emotion recognition performance, including mean accuracy of facial emotion recognition across all emotions (global level), sensitivity (*d’*), and response bias (*c*). We also collected reaction time (RT) data (time taken for the participant to indicate that they had recognized the target stimulus) and emotion labeling reflection time (time taken to select a response option), to obtain indirect information about emotional information processing. In line with earlier work with individuals with psychopathic personality disorders (34), we also asked participants to indicate how easy it was to make a decision, and recorded reflection times. Based on previous research on individuals in prison who had committed sexual offenses, we expected that forensic inpatients who have committed sexual offenses would exhibit lower overall sensitivity (*d’*) compared to forensic inpatients who have committed non-sexual offenses and community members. Moreover, we hypothesized that forensic inpatients who have committed sexual offenses would show a more conservative response bias (*c*) than the two other groups for classifying negative emotions e.g., sadness or fear.

## 2 Materials and methods

### 2.1 Participants

Required sample size was computed using G*Power software to detect the within-between interaction using repeated measures ANOVA (35). A total sample size of 36 (12 participants in each group) was necessary to achieve 95% power, with an effect size value of *f* = .25 (medium sized effect) (36). The sample consisted of 112 male participants, divided into three groups: 37 forensic inpatients who have committed sexual offenses, 25 forensic inpatients who have committed non-sexual offenses, and 50 community members. Forensic inpatients were recruited from the High-Risk Secure Forensic Hospital of “*Les Marronniers*” Psychiatric Regional Center in Tournai, Belgium. All inpatients were hospitalized under Belgian Law related to the internment of persons (37) as they were found Not Guilty by Reason of Insanity. Any potential participant with a visual and/or auditory impairment uncorrected by a medical device (glass, hearing device) was excluded. Forensic inpatients had to be assessed by their psychologist to confirm that they were well enough to participate and were able to read instructions and basic words such as “anger” or “fear”.

### 2.2 Instruments

#### 2.2.1 Demographics

A short socio-demographic questionnaire was administered to collect information such as age, gender, handedness, ethnicity, education years, and presence of any uncorrected visual and/or auditory impairment. For forensic inpatients, we consulted their official internment files to collect information relating to length of stay and criminal record. Based on the latter, we categorized forensic inpatients into two groups: forensic inpatients who had committed sexual offenses (must have committed at least one sexual offense), and forensic inpatients who had committed non-sexual offenses (criminal record must have shown no history of sexual offending).

#### 2.2.2 Mini International Neuropsychiatric Interview (MINI)

The MINI (38) is a structured clinical interview used to diagnose the presence of Major Mental Disorders (former Axis I), according to the DSM-IV-TR (39). This interview is composed of 17 independent modules (2 optional) categorized into six meta-modules: Mood Disorders (e.g., Major Depressive Episode, Dysthymia), Anxiety Disorders (e.g., Panic Disorder, Agoraphobia), Substance Use and Abuse, Psychotic Disorders, Post-Traumatic Disorder, and Eating Disorders (e.g. Anorexia Nervosa, Bulimia Nervosa). Two additional modules assess Major Depressive Episode with Melancholy Features and Antisocial Personality Disorder. MINI includes 120 dichotomous questions (yes/no) related to symptoms for each diagnosis. The French-validated version was used in this research (40). Forensic inpatients were assessed using the MINI by trained psychologists at least one month after they arrived at the High-Risk Secure Forensic Hospital. Scores were recorded from participants clinical case files. We used this version of the MINI as data collection began before the validation of the MINI for DSM-5. We decided to use this version to maintain the consistency of the data already collected. Previous research among forensic inpatients from the same High-Risk Secure Forensic Hospital highlighted strong inter-rater reliability (Cohen’s *κ* ≥.84) for different mental disorders assessed with this version of MINI (41).

#### 2.2.3 Structured Clinical Interview for DSM-IV Axis II Disorders (SCID-II)

The SCID-II (42) is a semi-structured interview used to assess Personality Disorders (former Axis II), according to the DSM-IV-TR (39). Twelve personality disorder diagnoses were assessed: (Cluster A) Schizotypal, Schizoid, and Paranoid; (Cluster B) Histrionic, Borderline, Narcissistic, and Antisocial; (Cluster C) Avoidant, Dependent, and Obsessive-Compulsive; and two more not clustered, Negativist and Depressive. Assessments were conducted in two steps. First, a 119-item self-report questionnaire with dichotomous (yes/no) answers was administered. Second, all positively scored items were further explored using a semi-structured interview, asking the participant to illustrate self-assessed symptoms with concrete examples. The French-validated version of the SCID-II (43) was used in this research. Forensic inpatients were assessed using the SCID-II by trained psychologists at least one month after they arrived at the High-Risk Secure Forensic Hospital. Scores were recorded from participants clinical case files. We used this version of the SCID as data collection began before the validation of the SCID-PD for DSM-5. We decided to use this version to maintain the consistency of the data already collected. Among forensic inpatient samples from the same High-Risk Secure Forensic Hospital, a previous study highlighted that Cohen’s *κ* computed were ≥.81 across all Clusters disorders (41).

#### 2.2.4 Positive and Negative Affect Schedule (PANAS)

The PANAS (44) is a 20-item self-report questionnaire used to assess participants current affective state, factorized in Positive (PA) and Negative (NA) Affect. Each item is scored using a five-point Likert scale (1 = Very Slightly or Not at All; 5 = Extremely). Total scores vary between 10 and 50 for each scale (PA, NA). Normative values have been established using data collected from undergraduate psychology students: PA (M = 29.70; SD = 7.20) and NA (M = 14.8; SD = 6.2). The two PANAS subscales exhibited sufficient internal consistency in the present sample (PANAS PA α = .75; PANAS NA α = .73).

#### 2.2.5 Stimuli

Facial expression stimuli were taken from the *NimStim Set of Facial Expressions* (26). This set includes 672 colorized, static images of male and female, multi-ethnic (Caucasian, Afro-descendant, Latino, and Asian) models expressing eight emotions, including neutrality, calm, and Ekman’s six ‘discrete’ emotions: anger, disgust, fear, happiness, sadness, and surprise. This research included only images showing Ekman’s six ‘discrete’ emotions and neutrality. Except for surprise, open and closed-mouth poses were selected for each emotion category. From this set, four “practice” stimuli and 87 “task” stimuli [(5 emotions * 2 genders * 4 ethnicities * 2 poses) + (1 emotion * 2 genders * 4 ethnicities) – 1 missing stimulus^1^] were selected based on the highest mean proportion correct scores from the validation sample (female: *M* = .85, *SD* = .14; male: *M* = .84, *SD* = .15). The interrater agreement of the static stimuli set from the validation sample is close to strong (κ = 0.79), comparable to previous stimuli sets (26). Similarly, test-retest reliability is close to excellent (*r* = 0.84) (26). *FantaMorph 5.0* software (45) was used to morph each emotion stimulus with neutral, over 30 frames. The resulting frames were dynamized to obtain 87 video-stimuli (refresh rate: 60Hz) of ten seconds duration, starting with a neutral expression (0s) leading to a prototypical (*apex*) emotion (10s).

### 2.3 Procedure

Forensic inpatients were either approached by their psychologists or were approached by the research team. Each participant signed an informed consent form in accordance with the Declaration of Helsinki and the General Data Protection Regulation (GDPR) (46) on protecting personal data. Feedback concerning their task performances was provided to participants at their request. On average, two meetings were organized with voluntary patients for this study. The aim of the first meeting was to explain the research objectives, ask participants to provide written consent, and complete the demographic questionnaire. This first meeting (+/- 2 hours) took place in a quiet room in the patient’s care unit. The aim of the second meeting (+/- 2 hours) was to assess the participant’s affective state (PANAS) and ask them to complete the experimental task (facial emotion recognition). This meeting took place in a quiet room in a separate care unit. Psychopathological characteristics of forensic inpatients were assessed by trained psychologists at least one month after they arrived at the High-Risk Secure Forensic Hospital. Psychopathological, medication, and length of stay data were collected by trained psychologists and psychiatrists from the High-Risk Secure Forensic Hospital. The criminological data were collected from the participants’ criminal records. Anonymized data were then made available to researchers at the Center of Research in Social Defense for coding and analysis. Community members were recruited using published calls for participants on social media (Facebook, Instagram) and in public places using flyers (supermarkets, doctors’ waiting rooms, gyms, etc.). Only one meeting was planned with members of the community.

The experimental task was run using *E-Prime 2.0* (47) on an HP ZBook15 (15.6 inches; 1920*1080; refresh rate: 60Hz). The distance between the screen and the participant was 65cm (visual angle: 17.06°*11.42°). NimStim dynamic expressions were presented across four stimulus blocks in a pseudorandomized order. Each block of trials was followed by a ‘break’ screen, except for the last block, which was followed by a ‘task finished’ screen. Within each block, trials began with a fixation cross (2,000ms), followed by a video stimulus (10,000ms) selected at random. Participants were instructed to press the space bar as fast as possible to indicate that they recognized the emotion. If no response was detected before the stimulus ended, the final frame (100% prototypical emotion) remained on the screen until participants pressed the space bar. Two questions followed each stimulus – ‘What emotion did you perceive?’, accompanied by six forced choice options, reflecting the six ‘discrete’ emotions, and ‘Was it easy or difficult to recognize?’, accompanied by a six-point Likert scale, from (1) ‘Very difficult’ to (6) ‘Very easy.’ Participants responded using a *Cedrus* response box (RB-730 model) placed on a 3D-printed reading support with an angle 20°.

### 2.4 Data analysis

Statistical analyses were carried out using *IBM Statistics Package for Social Sciences* (SPSS) version 26 (48). Data were prepared for analysis by first computing mean scores for accuracy and easiness, RT, and reflection times for emotion labeling and easiness across all emotions. Mean easiness, RT, and reflection times were also computed separately for each of the six discrete emotions. Second, we used SDT formulas to compute *d’* and *c* indices for each emotion (31, 32).

Descriptive statistics were calculated for age, years of education, length of stay, criminological history, psychiatric diagnoses, total IQ, medication, and PANAS scores. Comparisons on socio-demographic data were undertaken using Kruskal-Wallis’*H*, followed by pairwise Mann-Whitney’s *U*. Fischer’s exact test was used to analyze frequency data relating to participants’ psychiatric diagnoses, medication, and criminological history.

Test of normality, using the Shapiro-Wilk (N < 50) or Kolmogorov-Smirnov (N ≥ 50) test, showed that task outcomes were not normally distributed. Data transformations were largely unsuccessful for achieving a normal distribution. We therefore analyzed task outcome data using the non-parametric Kruskal-Wallis’ *H* test, followed by pairwise Mann-Whitney’s *U* test comparisons (Dunn-Bonferroni correction, *p*-value threshold: *p* = .016). For all comparisons, non-parametric (*r* = $\frac{z}{\sqrt{N}}$ ) or frequency (Cramer’s *V*) effect sizes were computed (49). The former were interpreted based on Cohen’s norms (50):.20 = small;.50 = moderate;.80 = large.

We also reported non-parametric zero-order correlations (Spearman ρ) between control variables (age, years of education, length of stay, psychiatric diagnoses, total IQ, medication, and PANAS scores) and dependent variables in each group (see **Supplementary Tables 1**-**4**).

## 3 Results

### 3.1 Descriptives statistics and preliminary results

Descriptive statistics for socio-demographic variables and psychological functioning are shown in **Table 1**. Community members were younger than forensic inpatients who had committed sexual offenses (*U* = 455.50, *p* ≤.001; *r* = .43), and forensic inpatients who had committed non-sexual offenses (*U* = 367.00, *p* ≤.005; *r* = .33), and had more years of education than forensic inpatients who had committed sexual offenses (*U* = 48.00, *p* ≤.001; *r* = .82), and forensic inpatients who had committed non-sexual offenses (*U* = 24.00, *p* ≤.001; *r* = .79). The two forensic groups did not differ in years of education or total IQ, but forensic inpatients who had committed sexual offenses were older (*U* = 326.00, *p* = .050; *r* = .25), and had been hospitalized for longer (*U* = 189.00, *p* ≤.001 *r* = .44) than forensic inpatients who had committed non-sexual offenses. The three groups did not differ in levels of positive and negative affective states.

**Table 1:** Descriptive and comparative analyses (Kruskal-Wallis’ *H* or Mann-Whitney’s *U*) regarding the socio-demographic variables and PANAS scores between the three groups.

	FICSOs		FICNSOs		CoM		Statistics	
	*n*	M (SD)	*n*	M (SD)	*n*	M (SD)	*H* or *U*	*p*
Age (years)	37	48.08 (14.51)	25	41.15 (10.78)	50	34.37 (14.18)	**20.00**	**≤.001**
Years of Education	37	5.03 (4.80)	25	6.36 (4.36)	50	15.44 (2.73)	**77.57**	**≤.001**
Total IQ (WAIS-IV)	21	75.90 (18.51)	17	74.88 (14.17)	N/A	N/A	169.50	.794
PANAS								
*PANAS – PA*	*37*	*31.73 (6.50)*	*25*	*30.92 (7.78)*	*50*	*31.58 (5.12)*	*0.25*	*.883*
*PANAS – NA*	*37*	*13.49 (3.40)*	*25*	*15.08 (5.75)*	*50*	*14.34 (4.83)*	*0.45*	*.797*

FICSOs, Forensic Inpatients who have Committed Sexual Offenses; FICNSOs, Forensic Inpatients who have Committed Non-Sexual Offenses; CoM, Community Members; PANAS – PA, PANAS – Positive Affect; PANAS – NA, PANAS – Negative Affect; N/A, Not Applicable. Bold values mean significant differences.

Data on psychopathology, medication, and forensic criminal histories of the two forensic groups are presented in **Table 2**. Forensic inpatients who had committed non-sexual offenses showed a higher prevalence of major mental disorders, especially psychotic and addiction disorders, and had more diverse criminal histories than forensic inpatients who had committed sexual offenses. A larger proportion of forensic inpatients who had committed non-sexual offenses were medicated with typical and atypical antipsychotics compared to forensic inpatients who had committed sexual offenses. Other medications, such as antiparkinsonian and substitution treatment, were also administered more frequently in this group.

**Table 2:** Descriptive and comparative analyses (Fischer exact test) regarding the psychopathology, medication, and criminological histories between the two forensic inpatient groups.

	FICSOs		FICNSOs		Statistics		
	*n*	% (*n*)	*n*	% (*n*)	$\chi^{2}$	*p*	Cramer’s *V*
Major Mental Disorders	34	35.30 (12)	25	80.00 (20)	**11.60**	***≤.001***	**.44**
*Mood Disorders*	*35*	*20.00 (7)*	*25*	*20.00 (5)*	*0.01*	*≥ .999*	
*Addiction Disorders*	*35*	*22.90 (8)*	*25*	*52.00 (13)*	***5.44***	***.028***	***.30***
*Psychotic Disorders*	*35*	*2.90 (1)*	*24*	*36.00 (9)*	***11.53***	***≤.001***	***.44***
*Anxious Disorders*	*34*	*11.80 (4)*	*25*	*12.00 (3)*	*0.01*	*≥ .999*	
*Eating Disorders*	*33*	*00.00 (0)*	*25*	*4.00 (1)*	*1.42*	*.417*	
*Paraphilic Disorders*	*36*	*50.00 (18)*	*N/A*	*N/A*	*N/A*	*N/A*	*N/A*
Personality Disorders	35	85.70 (30)	25	84.00 (21)	0.03	≥ .999	
*Cluster A – Odd, Eccentric*	*35*	*20.00 (7)*	*25*	*24.00 (6)*	*0.14*	*.758*	
*Cluster B – Dramatic, Emotional*	*35*	*74.30 (26)*	*25*	*80.00 (20)*	*0.27*	*.760*	
*Cluster C – Anxious, Fearful*	*35*	*37.10 (13)*	*25*	*20.00 (5)*	*2.04*	*.253*	
Axis I – Axis II Comorbidity	*35*	*54.30 (19)*	*25*	*80.00 (20)*	*4.24*	.056	
Medication							
*Anxiolytic and Sleep-Inducing*	*37*	*62.20 (23)*	*25*	*72.00 (18)*	*0.64*	*.585*	
*Typical Antipsychotic*	*37*	*32.40 (12)*	*25*	*68.00 (17)*	***7.58***	***.009***	***.35***
*Atypical Antipsychotic*	*37*	*48.60 (18)*	*25*	*92.00 (23)*	***12.52***	***≤.001***	***.45***
*Antidepressant*	*37*	*56.80 (21)*	*25*	*76.00 (19)*	*2.41*	*.177*	
*Anticonvulsant*	*37*	*18.90 (7)*	*25*	*36.00 (9)*	*2.27*	*.151*	
*Hormone Therapy*	*37*	*10.80 (4)*	*25*	*4.00 (1)*	*0.93*	*.640*	
*Other*	*37*	*13.50 (5)*	*25*	*44.00 (11)*	***7.24***	***.016***	***.34***
Current offenses							
*Current sexual offense*	*37*	*89.20 (33)*	*25*	*00.00 (0)*	***47.67***	***≤.001***	***.88***
*Current contact sexual offense*	*37*	*89.20 (33)*	*25*	*00.00 (0)*	***47.67***	***≤.001***	***.88***
*Current non-contact sexual offense*	*37*	*13.50 (5)*	*25*	*00.00 (0)*	*3.67*	*.076*	
*Current non-sexual offense*	*37*	*25.10 (13)*	*23*	*95.70 (22)*	***21.37***	***≤.001***	***.60***
*Current non-sexual violent offense*	*37*	*21.60 (8)*	*25*	*64.00 (16)*	***11.29***	***≤.001***	***.43***
*Current non-sexual non-violent offense*	*37*	*24.30 (9)*	*23*	*78.90 (18)*	***16.67***	***≤.001***	***.53***
Past offenses							
*Past sexual offense*	*37*	*43.20 (16)*	*25*	*00.00 (0)*	***14.57***	***≤.001***	***.48***
*Past contact sexual offense*	*37*	*43.20 (16)*	*25*	*00.00 (0)*	***14.57***	***≤.001***	***.48***
*Past non-contact sexual offense*	*37*	*16.20 (6)*	*25*	*00.00 (0)*	*4.49*	*.073*	
*Past non-sexual offense*	*37*	*51.40 (19)*	*25*	*72.00 (18)*	*2.64*	*.121*	
*Past non-sexual violent offense*	*37*	*27.00 (10)*	*25*	*48.00 (12)*	*2.87*	*.110*	
*Past non-sexual non-violent offense*	*37*	*48.60 (18)*	*25*	*72.00 (18)*	*3.34*	*.115*	

FICSOs, Forensic Inpatients who have Committed Sexual Offenses; FICNSOs, Forensic Inpatients who have Committed Non-Sexual Offenses. N/A, Not Applicable.

Bold values mean significant differences.

Associations between control and dependent variables indicate several significant correlations (see **Supplementary Tables 1**-**4**).

### 3.2 Comparative results

#### 3.2.1 Across all emotions

**Table 3** shows significant differences between groups in RT, accuracy, emotion labeling reflection time, and easiness, but not in easiness reflection time. Pairwise comparisons highlighted that despite performing similarly to each other, both forensic inpatient groups performed differently compared to community members.

**Table 3:** Emotion recognition scores (all emotions combined) for forensic inpatients who have committed sexual offenses, forensic inpatients who have committed non-sexual offenses, and community members.

	FICSOs (*n* = 37)	FICNSOs (*n* = 25)	CoM (*n* = 50)	Statistic	
Dependent Variables	M (SD)	M (SD)	M (SD)	*H*	*p*
Reaction Time (RT)	11254.02 (4007.46)	10476.59 (3339.33)	6455.09 (1560.53)	51.18	**≤.001**
Accuracy	.64 (.13)	.67 (.14)	.78 (.06)	35.91	**≤.001**
Emotion Labeling Reflection Time	3295.25 (3128.03)	3332.26 (1162.81)	1951.98 (470.89)	38.89	**≤.001**
Easiness Mean Score	4.61 (.91)	4.64 (.64)	3.87 (.53)	27.26	**≤.001**
Easiness Reflection Time	3117.86 (2144.56)	2864.02 (1776.45)	2074.49 (535.71)	4.50	.106

FICSOs, Forensic Inpatients who have Committed Sexual Offenses; FICNSOs, Forensic Inpatients who have Committed Non-Sexual Offenses; CoM, Community Members.

Bold values mean significant differences.

Both forensic groups took longer to recognize facial expressions of emotion (forensic inpatients who had committed sexual offenses: *U* = 182.00, *p* ≤.000, *z* = -6.379, *r* = .68; forensic inpatients who had committed non-sexual offenses: *U* = 148.00, *p* ≤.001, *z* = -5.361, *r* = .62), with a moderate effect size (*r* ≥.50), and showed worse accuracy (forensic inpatients who had committed sexual offenses: *U* = 297.50, *p* ≤.001, *z* = -5.393, *r* = .55; forensic inpatients who had committed non-sexual offenses: *U* = 252.00, *p* ≤.001, *z* = -4.200, *r* = .48), with either a small or moderate effect size, compared to community members. Emotion labeling reflection time results followed the same pattern as RT (forensic inpatients who had committed sexual offenses: *U* = 430.00, *p* ≤.001, *z* = -4.250, *r* = .45; forensic inpatients who had committed non-sexual offenses: *U* = 99.00, *p* ≤.001, *z* = -5.912, *r* = .68), with a small and moderate effect size. Counterintuitively, both forensic inpatients who had committed sexual offenses (*U* = 427.00, *p* ≤.001, *z* = -4.726, *r* = .51) and forensic inpatients who had committed non-sexual offenses (*U* = 232.50, *p* ≤.001, *z* = -4.412, *r* = .51) rated the task as easier compared to community members, with a moderate effect size. Again, no difference was found between forensic inpatients who had committed sexual offenses and forensic inpatients who had committed non-sexual offenses.

#### 3.2.2 Specific emotions

**Table 4** shows Kruskal-Wallis group comparison statistics for RT, sensitivity, response bias, emotion labeling reflection time, easiness, and easiness reflection time, with follow-up Mann-Whitney group comparisons (with Dunn-Bonferroni correction) described in the text.

**Table 4:** Emotion recognition scores (specific emotion) for forensic inpatients who have committed sexual offenses, forensic inpatients who have committed non-sexual offenses, and community members.

	FICSOs (*n* = 37)	FICNSOs (*n* = 25)	CoM (*n* = 50)	Statistic	
Dependent Variables	M (SD)	M (SD)	M (SD)	*H*	*p*
Anger					
Reaction Time (RT)	11153.07 (4557.36)	10100.03 (3437.45)	6567.10 (1720.09)	41.66	**≤.001**
*d’*	2.39 (.82)	2.63 (1.03)	2.91 (.49)	10.03	**.007**
*c*	0.35 (.48)	0.26 (.39)	0.24 (.28)	2.68	.262
Emotion Labeling Reflection Time	2596.82 (1365.38)	2830.19 (1293.43)	1842.32 (750.09)	19.37	**≤.001**
Easiness Mean Score	4.48 (1.16)	4.71 (.64)	3.79 (.69)	24.57	**≤.001**
Easiness Reflection Time	2985.21 (2149.30)	2772.44 (2040.49)	2074.49 (623.09)	2.33	.312
Disgust					
Reaction Time (RT)	10474.18 (4119.64)	9486.76 (3337.72)	6278.71 (1738.43)	37.64	**≤.000**
*d’*	1.73 (.93)	1.90 (.77)	2.31 (.50)	11.88	**.003**
*c*	0.76 (.54)	0.63 (.36)	0.69 (.30)	.66	.717
Emotion Labeling Reflection Time	2628.03 (1337.57)	2879.48 (950.78)	1714.68 (508.14)	35.07	**≤.001**
Easiness Mean Score	4.59 (1.01)	4.53 (.68)	3.83 (.64)	22.78	**≤.001**
Easiness Reflection Time	3084.95 (2192.13)	2954.58 (2025.32)	2122.21 (620.42)	2.45	.293
Happiness					
Reaction Time (RT)	9374.96 (5381.51)	8355.43 (2901.46)	4854.88 (1230.36)	54.48	**≤.001**
*d’*	3.57 (.65)	3.48 (.79)	3.99 (.34)	16.73	**≤.001**
*c*	0.20 (.28)	0.27 (.33)	0.24 (.16)	1.56	.459
Emotion Labeling Reflection Time	3053.87 (1531.14)	3053.08 (1345.54)	1700.13 (461.18)	35.76	**≤.001**
Easiness Mean Score	4.92 (.87)	4.91 (.66)	4.60 (.60)	8.52	**.014**
Easiness Reflection Time	3093.65 (2535.93)	2809.75 (1760.81)	1911.59 (526.81)	6.05	**.048**
Fear					
Reaction Time (RT)	12196.36 (4562.82)	11437.50 (3876.14)	6792.50 (1774.60)	49.59	**≤.001**
*d’*	1.30 (.60)	1.51 (.72)	2.22 (.62)	35.94	**≤.001**
*c*	0.99 (.53)	0.96 (.40)	0.85 (.62)	1.68	.431
Emotion Labeling Reflection Time	3543.40 (2450.56)	3879.58 (1602.77)	2294.51 (850.97)	24.30	**≤.001**
Easiness Mean Score	4.51 (.98)	4.50 (.75)	3.66 (.64)	26.98	**≤.001**
Easiness Reflection Time	3342.36 (2336.94)	3056.35 (1913.04)	2213.31 (606.38)	5.76	.056
Surprise					
Reaction Time (RT)	11397.76 (3920.05)	11375.57 (4656.04)	6320.37 (1690.30)	53.40	**≤.001**
*d’*	1.65 (.78)	1.84 (.86)	2.51 (.57)	27.20	**≤.001**
*c*	0.68 (.68)	0.51 (.62)	0.21 (.33)	12.75	**.002**
Emotion Labeling Reflection Time	3094.55 (1879.75)	3828.20 (2031.76)	1854.81 (628.77)	32.98	**≤.001**
Easiness Mean Score	4.68 (.99)	4.69 (.66)	4.08 (.64)	20.46	**≤.001**
Easiness Reflection Time	2840.73 (1756.86)	2584.71 (2010.18)	1920.92 (550.35)	6.30	**.043**
Sadness					
Reaction Time (RT)	13051.46 (4196.26)	12613.80 (4085.25)	7870.73 (1950.48)	48.14	**≤.001**
*d’*	1.78 (.71)	1.88 (.74)	2.46 (.43)	28.35	**≤.001**
*c*	0.64 (.47)	0.68 (.39)	0.54 (.30)	2.40	.301
Emotion Labeling Reflection Time	4766.66 (7778.23)	3805.19 (1525.03)	2278.27 (835.95)	27.84	**≤.001**
Easiness Mean Score	4.19 (.95)	4.53 (.77)	3.36 (.57)	42.25	**≤.001**
Easiness Reflection Time	3232.81 (2267.67)	2878.63 (1554.08)	2136.32 (618.97)	4.97	.083

FICSOs, Forensic Inpatients who have Committed Sexual Offenses; FICNSOs, Forensic Inpatients who have Commited Non-Sexual Offenses; CoM, Community Members; H, Kruskal-Wallis’ H.

Bold values mean significant differences.

##### 3.2.2.1 Reaction Time (RT)

Forensic inpatient groups showed significantly longer RTs than community members across all emotions. We observed moderate effect sizes for differences in RT between forensic inpatients who had committed sexual offenses and community members, for anger (*U* = 243.00, *p* ≤.001, *z* = -5.855, *r* = .63), disgust (*U* = 256.00, *p* ≤.001, *z* = -5.744, *r* = .61), happiness (*U* = 164.00, *p* ≤.001, *z* = -6.534, *r* = .70), fear (*U* = 192.00, *p* ≤.001, *z* = -6.293, *r* = .67), surprise (*U* = 136.00, *p* ≤.001, *z* = -6.774, *r* = .73), and sadness (*U* = 210.00, *p* ≤.001, *z* = -6.139, r = .66).

For the comparison between forensic inpatients who had committed non-sexual offenses and community members, we observed a small-sized effect for disgust (*U* = 256.00, *p* ≤.001, *z* = -4.147, *r* = .48), with moderate effects sizes observed for anger (*U* = 207.00, *p* ≤.001, *z* = -4.698, *r* = .54), happiness (*U* = 126.00, *p* ≤.001, *z* = -5.608, *r* = .65), fear (*U* = 157.00, *p* ≤.001, *z* = -5.260, *r* = .61), surprise (*U* = 168.00, *p* ≤.001, *z* = -5.136, *r* = .59), and sadness (*U* = 156.00, *p* ≤.001, *z* = -5.271, *r* = .60).

##### 3.2.2.2 Sensitivity (d’)

Unlike RT results, group differences in sensitivity (*d’)* were more subtle. Across all emotion categories except for disgust, forensic inpatient groups showed lower sensitivity compared to community members but performed at a similar level to each other. We observed moderate effect sizes for comparisons of forensic inpatients who had committed sexual offenses with community members for fear (*U* = 272.50, *p* ≤.001, *z* = -5.604, *r* = .60), surprise (*U* = 337.00, *p* ≤.001, *z* = -5.050, *r* = .54), and sadness (*U* = 350.00, *p* ≤.001, *z* = -4.934, *r* = .53), with small effect sizes observed for anger (*U* = 548.00, *p* ≤.001, *z* = -3.239, *r* = .35), disgust (*U* = 546.50, *p* ≤.001, *z* = -3.250, *r* = .35), and happiness (*U* = 550.00, *p* ≤.001, *z* = -3.295, *r* = .35). Comparisons between forensic inpatients who had committed non-sexual offenses and community members revealed small effect sizes for fear (*U* = 284.00, *p* ≤.001, *z* = -3.835, *r* = .44), happiness (*U* = 318.50, *p* ≤.001, *z* = -3.520, *r* = .41), surprise (*U* = 350.00, *p* ≤.005, *z* = -3.086, *r* = .36), and sadness (*U* = 298.50, *p* ≤.001, *z* = -3.672, *r* = .42).

##### 3.2.2.3 Response bias (c)

Response bias did not vary between groups for any of the emotion categories, except surprise, where forensic inpatients who had committed sexual offenses showed a more conservative response style compared to community members (*U* = 499.00, *p* ≤.001, *z* = -3.658, *r* = .39).

##### 3.2.2.4 Emotion labeling reflection time

Results for emotion labeling reflection time followed the same overall pattern observed for RT, with both forensic inpatient groups showing longer reflection times than community members. As with RT, the two forensic inpatient groups exhibited similar performance across all emotions.

Small-sized effects were observed for differences between forensic inpatients who had committed sexual offenses and community members for anger (*U* = 518.00, *p* ≤.001, *z* = -3.494, *r* = .37), disgust (*U* = 452.00, *p* ≤.001, *z* = -4.061, *r* = .43), fear (*U* = 562.00, *p* ≤.005, *z* = -3.117, *r* = .33), surprise (*U* = 544.00, *p* ≤.001, *z* = -3.271, *r* = .35), and sadness (*U* = 462.00, *p* ≤.001, *z* = -3.975, *r* = .43), while a large effect size was observed for time to label happy expressions (*U* = 378.00, *p* ≤.001, *z* = -4.696, *r* = .50).

Conversely, we observed large effect sizes for differences between forensic inpatients who had committed non-sexual offenses and community members, for disgust (*U* = 133.00, *p* ≤.001, *z* = 5.530, *r* = -.64), happiness (*U* = 157.00, *p* ≤.001, *z* = -5.260, *r* = .64), fear (*U* = 205.00, *p* ≤.001, *z* = -4.720, *r* = .54), surprise (*U* = 108.00, *p* ≤.001, *z* = -5.811, *r* = .67), and sadness (*U* = 205.00, *p* ≤.001, *z* = -4.720, *r* = .54), while a small effect size was observed for differences in labeling angry expressions (*U* = 295.00, *p* ≤.001, *z* = -3.709, *r* = .43).

##### 3.2.2.5 Easiness response

Easiness ratings showed the inverse pattern of results observed for sensitivity (*d’*), with both forensic inpatient groups rating the task of classifying emotions as significantly easier than community members. There was no difference in ease of classification between the two forensic inpatient groups. Small effect sizes were observed for the difference between forensic inpatients who had committed sexual offenses and community members for anger (*U* = 517.50, *p* ≤.001, *z* = -3.501, *r* = .37), disgust (*U* = 456.00, *p* ≤.001, *z* = -4.030, *r* = .43), happiness (*U* = 625.00, *p* ≤.010, *z* = -2.574, *r* = .27), fear (*U* = 419.00, *p* ≤.001, *z* = -4.347, *r* = .47), and surprise (*U* = 472.00, *p* ≤.001, *z* = -3.897, *r* = .42), with a large effect size for ease of labeling sad expressions (*U* = 290.00, *p* ≤.001, *z* = -5.455, *r* = .58).

Forensic inpatients who had committed non-sexual offenses rated the task easier than community members (except for happy expressions), with small effect sizes for disgust (*U* = 281.50, *p* ≤.001, *z* = -3.864, *r* = .45), fear (*U* = 244.00, *p* ≤.001, *z* = -4.286, *r* = .49), and surprise (*U* = 308.00, *p* ≤.001, *z* = -3.570, *r* = .41), and moderate effect sizes for anger (*U* = 204.50, *p* ≤.001, *z* = -4.732, *r* = .54), and sadness (*U* = 150.50, *p* ≤.001, *z* = -5.338, *r* = .62).

##### 3.2.2.6 Easiness reflection time

Group differences for easiness reflection time were largely non-significant, with no significant differences between groups for any of the emotion expressions.

## 4 Discussion

Socio-affective functioning, referring to how people interact with others, represents one of the four domains of dynamic risk for sexual recidivism in the Structured Assessment of Risk and Needs (4). However, relatively little work has been carried out to understand emotion processing in people who have sexually offended, with most of the work that has taken place focusing on recognition of other’s emotional facial expressions. Previous studies have investigated emotion recognition exclusively among individuals in prison who have committed sexual offenses (14), and have used a diverse set of methodological designs, and recruited heterogeneous samples, leading to contradictory results. This study aimed to investigate recognition of the six ‘discrete’ facial expressions, identified by Ekman and Friesen (51), among forensic inpatients who had committed sexual offenses, using SDT to examine sensitivity and response bias (*d’* and *c*). In addition, we also collected reaction times, emotion labeling, and easiness reflection times, as well as the participants’ perceived easiness for categorizing the stimuli, allowing for the most comprehensive assessment of emotion recognition in this population to date.

In terms of accuracy and sensitivity, overall, our results show that the forensic inpatients who had committed sexual offenses were less accurate and less sensitive than community members, both across all emotions and within each emotion category: anger, disgust, happiness, fear, sadness, and surprise. Our results partially support previous findings that have identified more global problems in the processing of emotional facial expressions among individuals in prison in comparison to community members (16, 17). Other studies have also identified problems in emotion recognition, but these have tended to be restricted to particular emotion categories, such as anger (12, 15), disgust (12, 15, 20, 21), and fear (12, 15, 19). Our two forensic inpatient groups did not differ in accuracy or sensitivity, suggesting that problems in recognizing others’ emotional expressions are associated with severe antisocial behavior more generally, rather than limited to sexual offending (52). Although others have found differences between individuals who have committed sexual offenses and those who have committed non-sexual offenses (15, 17, 21), these differences may be accounted for, at least in part, by methodological differences. For example, past studies have used static stimuli, which provide less information for the observer about the dynamic unfolding of the emotional expression (29).

In contrast to forensic inpatients who had committed sexual offenses, forensic inpatients who had committed non-sexual offenses showed lower sensitivity than community members to emotional expressions, with the exception of anger and disgust. These findings are in line with previous meta-analyses that highlighted intact anger (52, 53) and disgust (53) recognition among individuals with antisocial or psychopathic personality disorders. Indeed, antisocial populations tend to exhibit a ‘hostile attribution bias’ or the tendency to attribute anger to ambiguous stimuli or when lacking contextual information (see (54) for a review). This information processing bias is thought to be linked with adverse childhood experiences and insecure attachment (54), and is considered a mediating factor in the relationship between facial affect recognition and aggression among aggressive individuals (55).

The three groups exhibit similar performance for *c*, with no group showing a particular preference for a particular emotion category label, with the exception of surprise. Forensic inpatients who had committed sexual offenses were less likely to select surprise than community members. Expressions such as happiness, fear, or anger, are typically associated with a particular valence (positive/pleasant or negative/unpleasant), but the location of surprise on this axis is uncertain (56). Some authors even consider it not as an emotion, *per se*, but rather as a pre-emotion, an “*emotional chameleon*” (p.2) (56), while others have suggested that surprise is more negative than positive (56, 57). The expression of surprise also bears physical similarities to the expression of fear, with information from the eyes and information from the mouth used to transmit both expressions (58). It is perhaps unsurprising therefore that these two emotions are often confused by community members (59). Thus, it may be speculated that the more conservative use of surprise in forensic inpatients who had committed sexual offenses reflects a preference for other emotion labels to the more ambiguous label of surprise.

The results of our study also highlight that the two forensic inpatient groups took significantly longer to recognize emotions (across all emotions and for each emotion category) and to select an emotion category label (e.g., ‘anger,’ ‘disgust,’ etc.). However, there were no differences compared to community members in the time taken to rate the easiness of the task. To the authors’ knowledge, no previous studies have investigated reaction and reflection times related to emotion recognition among individuals in prison or forensic inpatients who had committed sexual offenses. Indeed, similar work has tended to rely on accuracy and/or hit rates alone (60). In normative populations, findings indicate that happiness is the fastest emotion labeled, reflecting the relative uniqueness with which the expression is transmitted (58), followed by anger and fear (60–62). In clinical populations, a recent review (63) has shown that patients with bipolar disorders tend to take longer than healthy controls to recognize emotions, overall, especially anger or happiness, whereas others suggest faster processing of negative emotional cues such as anger, disgust, or sadness. Patients diagnosed with schizophrenia also take longer to label emotional expressions compared to control samples (64). These findings are partially supported by the results reported here, with forensic inpatients who had committed non-sexual offenses (but not those who had committed sexual offenses) requiring longer processing times compared to community members.

Lastly, an interesting result of this study concerns the perception of the task, which was considered easier by both forensic inpatient groups compared to the community members, a pattern that was the inverse of actual task performance. No difference was found between the three groups regarding the time needed to rate easiness. One of the few studies that has assessed perceptions of task difficulty during emotional face processing in an offending population was conducted with inmates with psychopathic personality disorder (34). In that study, a similar pattern of performance was reported, with individuals in prison reporting greater easiness despite poorer task performance. This apparent disconnect between perceived and actual performance may have implications for therapeutic approaches that aim to improve socio-affective function, and it may be important for individuals to develop insight into their own emotion processing competencies to improve awareness and understanding of others.

Despite some limitations, this study helps to lay foundations for future research. One limitation of the current work relates to the absence of matching of experimental and community groups, and the heterogeneous nature of forensic inpatients. Future work should aim to match groups on socio-demographic variables such as age and years of education, but also length of stay, total IQ, mental disorders, and medication. This is especially important considering work showing that emotion recognition impairments that are characteristic of people with psychopathy better reflect a deficit in general mental ability (65). Findings suggesting that psychopaths’ impaired emotion recognition is better accounted for by general mental ability challenge theories of emotion recognition in psychopathic personality, and also highlight the importance of understanding confounding factors. Further, due to the restricted statistical power and distribution of the data, we were unable to use parametric tests, including ANCOVAs, to test interactions that have been reported by others, including between Emotion*Group*Gender. In our study, age, length of stay, psychiatric diagnoses, and prescribed medications were all associated with outcome measures and could, therefore, have influenced the pattern of results. For example, systematic literature reviews and meta-analyses identify associations between substance use disorder (66) or Cluster A disorders, especially schizotypal (67), with poorer facial emotion recognition task performance.

Future research should aim to develop a more nuanced understanding of emotion recognition abilities in people who have sexually offended. People with convictions for sexual offenses are a highly heterogenous population who can be distinguished based on simple (e.g., adult versus child victims, contact versus online offenses) or more complex psychological profiles based on patterns of dynamic risk (2). For example, comparisons between people with different victim histories or victim preferences (e.g., children versus adults, stranger versus acquaintance) could help to confirm or challenge recent findings showing no difference in accuracy between people with convictions for sexual offenses with and without child victims (22). This question was beyond the scope of this study, and sample composition in terms of victimology precluded such analyses. Future work should also examine whether impairments in emotion recognition in people who have committed sexual offenses are associated with a sexual interest in children (i.e., a diagnosis of pedophilia). Further, comparing performance when people view adult versus child stimuli seems crucial, and could help to elucidate whether heightened emotional congruence with children is associated with greater sensitivity to children’s emotional expressions, with children representing non-threatening privileged social interlocutors (68). In addition, in general population samples women tend to show slightly superior task performance compared to men when asked to recognize non-verbal emotions, especially for negative emotions such as anger (69). Comparisons between male and female forensic inpatients who have committed sexual or non-sexual offenses and relevant control groups could be revealing about the specificity of emotion recognition impairments to men who have committed sexual offenses, and differences in socio-affective function associated with sexual offending in men and women (70).

Whereas therapeutic programs aim to prevent sexual recidivism by reducing areas of risk, they also consider other underlying processes and aim to help people who have committed sexual offenses to develop techniques that aid, for example, emotion regulation and self-management. Theoretical models that inform risk management and intervention programs have identified self-regulation, especially of negative emotions, as a key construct (71, 72) or even a causal factor (73) in the etiology of sexual offending. Being able to recognize negative emotions accurately is a necessary but not sufficient condition to enroll in a restorative justice pathway (74). It enables the transformation process through the expression, and therefore recognition and regulation, of negative emotions such as remorse and guilt. This therapeutic target is central in the care pathway as forensic inpatients who have committed sexual offenses exhibit a pervasive deficit in emotion recognition compared to community members.

## 5 Conclusion

This study, being the first to assess dynamic facial emotion recognition competencies among forensic inpatients who have committed sexual offenses in French-speaking Belgium, showed that this group exhibited lower levels of performance than community members in accuracy and sensitivity, and provided support for more general impairments rather than difficulties that are limited to particular emotions.

## Data availability statement

The datasets presented in this article are not readily available because Ongoing research in a PhD thesis. However, the authors remain available for information and collaboration. Requests to access the datasets should be directed to LT, luca.tiberi@umons.ac.be.

## Ethics statement

Ethical approval was granted in 2019 by the Ethical Committee from the Faculty of Psychology and Educational Sciences of the University of Mons (Ref. *UMONS-2019.11.22-TL-001*) and by the Psychiatric Regional Center “*Les Marronniers*” Ethic Committee (Ref. *DV/VJ/PB/2019*). Researchers followed ethical recommendations from the Helsinki Declaration and the European General Data Protection Regulation (UE 2016/679) regarding data collection and storage. The participants provided their written informed consent to freely participate in this study.

## Author contributions

LT: Conceptualization, Data curation, Formal analysis, Investigation, Methodology, Writing – original draft, Writing – review & editing, Visualization. SG: Writing – review & editing. XS: Writing – review & editing, Formal analysis. AV: Supervision, Writing – review & editing, Project administration. TP: Supervision, Writing – review & editing, Project administration.

## Footnotes

1 Missing stimulus originates from NimStim database
