# Supplementary material for: Recognition of dynamic facial expressions of emotions in forensic inpatients who have committed sexual offenses: a signal detection analysis
Source: Front Psychiatry. 2024 Jun 13;15:1384789. doi: 10.3389/fpsyt.2024.1384789 (PMC11210403; doi:10.3389/fpsyt.2024.1384789)
Supplement: Supplementary file 1 [file Table_1.pdf]

Supplementary Material

Recognition of Dynamic Facial Expressions of Emotions in Forensic Inpatients Who Have Committed Sexual Offenses: A Signal Detection Analysis

Luca A. Tiberi*, Steven M. Gillespie, Xavier Saloppé, Audrey Vicenzutto, Thierry H. Pham

*** Correspondence:** Corresponding Author: luca.tiberi@umons.ac.be

Overall, age is positively and moderately associated with either reaction or reflection times, mainly among forensic inpatients who have committed sexual offenses (ρ = .37 – .52) and community members with a lower strength (ρ = .28 – .34). A pattern of negative and large associations (ρ = -.45 – -.80) is found between total IQ and easiness among forensic inpatients who have committed sexual offenses. Length of stay is principally positively and moderately correlated (ρ = .33 – .45) with reflection time, whereas it is negatively and moderately correlated with mean accuracy across all emotions (ρ = -.42), especially for disgust (ρ = -.50) and surprise (ρ = -.35) among forensic inpatients who have committed sexual offenses. There was no pattern concerning the length of stay among forensic inpatients who have committed non-sexual offenses. Significant and negative large associations are retrieved between the PANAS positive scale and the reaction and reflection times among forensic inpatients who have committed sexual offenses (ρ = -.33 – -.58) and, to a lesser extent, among community members (ρ = -.35 – -.53).

Further, we observe several negative and moderate correlations (ρ = -.34 – -.40) between psychiatric diagnoses and RT among forensic inpatients who have committed sexual offenses, especially with Cluster A personality disorders (paranoiac and schizoid). There were fewer correlations found among

forensic inpatients who have committed non-sexual offenses. However, a similar pattern of negative but large associations (ρ = -.54 – -.57) is found with another Cluster A personality disorder, schizotypal, with RT. Finally, concerning medication, two patterns are mainly found. On the one hand, there were positive and moderate correlations between antidepressants and easiness reflection time (ρ = .33-.47) among the two forensic inpatient groups, with a slightly stronger association for the forensic inpatients who have committed non-sexual offenses. On the other hand, negative and mainly large correlations (ρ = -.44 – -.60) between other medications (e.g., Ritalin, antischizophrenic) with RT were found only among forensic inpatients who have committed non-sexual offenses.

Supplementary Table 1 – Non-parametric (Spearman's ρ) correlations between socio-demographic characteristics and facial expressions of emotions recognition scores among the forensic inpatients who have committed sexual offenses, forensic inpatients who have committed non-sexual offenses, and community members.

	FICSOs (n = 37)						FICNSOs (n = 25)						CoM (n = 50)			
	**Age**	**YoE**	**LoS**	**IQ (n = 21)**	**PAP**	**PAN**	**Age**	**YoE**	**LoS**	**IQ (n = 17)**	**PAP**	**PAN**	**Age**	**YoE**	**PAP**	**PAN**
**All emotions combined**																
Reaction Time (RT)	.04	-.15	-.08	-.21	**-.50*** **	.05	-.21	.21	-.27	-.04	**.43***	-.18	**.28***	**.32***	-.06	**-.31***
Mean Accuracy	**-.49****	-.07	**-.42****	-.09	-.04	-.22	-.10	.33	-.03	.45	-.28	-.02	.12	.08	.04	-.24
Emotion Labeling Reflection Time (RfT)	**.37*****	-.26	**.42***	-.36	-.24	-.22	**.42***	-.31	-.01	.30	.25	**-.50***	**.34***	.05	-.07	.05
Easiness response	-.13	-.03	-.03	**-.60****	.27	-.29	.34	.01	-.06	-.26	.19	.02	-.07	-.20	.14	.01
Easiness Reflection Time (RfT)	**.48*****	-.15	.29	.07	-.32	.07	-.09	-.01	.19	.09	-.07	-.27	.17	.08	**-.37****	-.06
**Anger**																
Reaction Time (RT)	-.02	-.10	-.12	-.13	**-.48*****	.08	-.16	.24	-.25	.19	.36	-.32	**.38****	**.39****	-.04	**-.34***
d'	**-.43****	-.34	-.03	-.07	-.08	-.17	-.32	.37	-.17	.39	-.11	-.01	.06	.10	-.01	**-.31***
c	-.23	-.33	.25	.12	-.26	.12	.02	-.34	-.07	-.36	.30	-.15	.17	.13	.01	.04
Emotion Labeling Reflection Time (RfT)	.18	.28	-.12	-.35	**-.34***	-.16	**.46***	-.12	-.01	.25	.05	**-.47***	**.34***	.11	.05	-.05
Easiness response	-.08	-.01	-.01	**-.49***	.26	-.24	.25	-.02	.05	-.15	.16	.09	-.23	-.25	.08	.12
Easiness Reflection Time (RfT)	**.41***	.30	-.07	.17	**-.33***	.09	-.08	.16	.30	.21	-.21	-.34	.25	.11	**-.35***	.01
**Disgust**																
Reaction Time (RT)	.05	-.11	-.09	-.06	**-.49*****	.09	-.07	.22	-.21	.06	.29	-.25	.33	**.35***	-.10	**-.34***
d'	**-.42****	-.08	**-.50*****	.05	-.25	.05	-.03	.35	.12	.16	.01	-.23	**.31***	-.06	.04	-.14
c	**.40***	.02	**.45*****	.27	.14	.01	-.10	-.01	.02	-.01	.06	.05	.06	-.08	-.10	.04
Emotion Labeling Reflection Time (RfT)	.28	.12	**.34***	-.33	-.25	-.13	.30	-.16	.03	.17	.13	**-.51****	**.29***	.13	-.01	.06
Easiness response	-.05	-.14	.05	**-.58*****	.31	-.29	.36	-.11	-.10	-.31	.16	.06	-.14	**-.32***	.14	.08
Easiness Reflection Time (RfT)	**.41***	-.10	.29	.01	-.30	-.01	-.21	.13	.23	.10	-.11	-.09	.18	.20	**-.42*****	-.06
**Happiness**																
Reaction Time (RT)	.08	-.23	-.01	-.22	**-.58*****	.02	-.17	.26	-.31	.03	.20	-.28	.11	.22	-.05	-.16
d'	**-.35***	.13	-.29	-.06	-.04	-.21	.04	.21	.26	.28	-.02	-.02	-.07	.14	-.24	-.26
c	-.05	**-.41***	.05	.14	-.02	.07	.27	.31	**.44***	.11	-.38	-.38	-.19	.02	-.16	.26
Emotion Labeling Reflection Time (RfT)	**.41***	-.23	**.36***	.07	-.28	.01	.24	-.21	.10	.17	-.09	-.09	**.28***	.01	-.09	-.01
Easiness response	-.09	-.11	.07	**-.46***	**.33***	-.29	.10	.03	-.01	-.19	.26	.26	.01	.01	.18	-.01
Easiness Reflection Time (RfT)	**.44****	-.18	.26	.02	**-.34***	.08	-.06	-.27	.16	-.11	.04	.04	.07	.10	**-.53*****	.01
**Fear**																
Reaction Time (RT)	-.04	-.12	-.10	-.21	**-.52*****	.12	-.24	.25	-.23	.03	**.42***	-.11	.27	**.36****	-.11	-.28
d'	-.27	-.07	-.11	-.03	.09	-.17	-.23	.20	**-.42***	.37	-.11	.21	.17	.17	.09	-.12

c	.22	.14	-.02	.16	.04	.16	-.22	-.12	**.41***	.01	-.22	-.14	.09	.07	-.23	.03
Emotion Labeling Reflection Time (RfT)	.28	-.27	**.43****	-.14	-.11	-.18	.32	-.32	.01	.18	.18	-.38	.27	-.03	-.04	.07
Easiness response	-.14	-.03	-.04	**-.60****	.24	-.26	**.41***	-.09	-.12	-.40	.30	.05	.04	-.24	.19	-.04
Easiness Reflection Time (RfT)	**.48*****	-.09	.29	.18	-.30	.16	.01	.01	.19	.18	-.14	-.16	.14	.03	-.27	-.03
**Surprise**																
Reaction Time (RT)	.09	-.18	.05	-.23	**-.43****	-.03	-.08	.03	-.28	-.01	**.42***	-.11	**.29***	.25	-.03	-.24
d'	**-.34***	-.01	**-.35***	.14	.04	-.04	-.09	**.43***	.41	.33	**-.42***	-.13	-.03	.07	-.14	-.06
c	.25	.11	.25	-.18	.10	-.08	.09	-.38	-.20	-.25	.21	.17	-.08	-.08	.19	-.08
Emotion Labeling Reflection Time (RfT)	**.41***	-.23	**.46*****	-.14	-.08	-.18	.23	-.25	-.18	.11	.38	-.19	.23	-.17	-.08	-.01
Easiness response	-.21	-.13	-.06	**-.80*****	.16	-.29	.26	.04	.03	-.38	.32	.05	-.02	-.05	.20	.12
Easiness Reflection Time (RfT)	**.52*****	-.10	**.35***	.17	-.31	.04	-.04	-.18	-.07	.17	-.05	-.28	.17	.07	-.26	-.11
**Sadness**																
Reaction Time (RT)	-.10	-.19	-.15	-.39	**-.40***	-.01	-.18	.07	-.26	-.09	**.57*****	-.08	.25	.24	-.02	-.25
d'	-.20	-.14	-.25	-.10	-.04	-.08	-.01	.23	.15	**.53***	-.36	-.11	.08	.02	.01	-.19
c	.11	-.22	.16	.04	.07	.18	.13	-.03	-.20	-.26	.23	.17	-.20	-.09	.14	-.01
Emotion Labeling Reflection Time (RfT)	.31	**-.33***	**.33***	**-.45***	-.26	-.18	.30	-.07	.14	.21	.38	**-.40***	.19	.08	-.01	-.06
Easiness response	-.15	.01	.04	**-.52***	.21	-.21	**.42***	.01	-.11	-.24	.30	-.05	-.01	-.20	.10	.06
Easiness Reflection Time (RfT)	**.51*****	-.16	.33	.14	-.27	.04	-.02	-.13	.05	.09	-.07	-.32	.07	-.08	-.26	-.03

*Note: FICSOs = Forensic Inpatients who have Committed Sexual Offenses; FICNSOs = Forensic Inpatients who have Committed Non -Sexual Offenses; CoM = Community Members; A = Age, YoE = Years of Education; PAP = PANAS – Positive Affect; PAN = PANAS – Negative Affect; *p ≤.05; **p ≤.01; ***p ≤.005*

Supplementary Table 2 – Non-parametric (Spearman’s ρ) correlations between psychiatric diagnoses and facial expressions emotion recognition scores among the forensic inpatients who have committed sexual offenses (*n* = 37).

	FICSOs (*n* = 37)															
	**Mood**	**SUD**	**Psy**	**Anx**	**Avoi**	**Depe**	**Ob-Co**	**Pa-Ag**	**Dep**	**Para**	**Schy**	**Schi**	**His**	**Narc**	**Bor**	**Anti**
**All emotions combined**																
Reaction Time (RT)	.20	-.20	.22	.02	.02	.19	-.29	-.04	-.05	**-.37***	-.12	**-.37***	.24	.04	-.14	-.13
Mean Accuracy	-.02	.26	.07	-.24	.01	-.17	.01	-.12	-.23	-.17	.21	.15	-.07	-.10	.13	-.08
Emotion Labeling Reflection Time (RfT)	.09	-.11	-.08	.10	.19	-.07	-.27	.05	.27	.10	-.23	-.16	.27	.17	.01	.24
Easiness response	-.21	.05	-.07	-.06	-.30	-.19	.04	.04	-.06	.13	-.30	-.10	-.02	.12	-.31	.08
Easiness Reflection Time (RfT)	.23	**-.34***	.17	.10	.20	.09	-.10	-.04	.23	-.19	-.16	**-.39***	.27	.04	-.01	.03
**Anger**																
Reaction Time (RT)	.26	-.12	.20	.11	.01	.23	-.27	-.06	-.04	**-.34***	-.15	**-.38***	.24	.06	-.07	-.16
d’	.20	.26	.22	-.08	.05	.07	.09	.12	-.11	-.06	.16	.10	-.20	-.01	.30	-.04
c	-.01	-.05	-.08	.08	.07	.13	-.17	-.06	.04	.12	-.06	.19	.14	.06	-.03	-.18
Emotion Labeling Reflection Time (RfT)	.24	-.16	.10	.08	.18	.09	-.26	.11	.27	.10	-.33	-.15	.29	.16	.07	.10
Easiness response	-.26	.04	-.14	-.10	-.29	-.18	.01	.09	-.07	.12	-.31	-.14	.04	.04	**-.38***	.03
Easiness Reflection Time (RfT)	.25	**-.37***	.25	.15	.28	.19	-.02	.08	.24	-.10	-.11	**-.40***	.27	.08	.04	-.01
**Disgust**																
Reaction Time (RT)	.15	-.22	.17	.09	-.01	.18	-.18	-.05	.01	-.33	-.13	**-.37***	.22	.01	-.17	-.03
d’	.03	.11	.17	-.10	.01	-.04	-.10	-.16	-.21	-.26	.16	.20	-.17	-.10	.04	.07
c	-.13	-.04	-.17	.14	-.16	-.04	.31	.27	.24	.10	-.04	-.15	-.05	.19	-.17	.16
Emotion Labeling Reflection Time (RfT)	.01	-.13	.08	.15	.07	.05	-.03	.18	.28	.27	-.24	-.10	.29	.18	.01	.13
Easiness response	-.12	.06	-.03	.02	-.22	-.19	.09	.01	-.01	.12	**-.34***	-.17	-.11	.20	-.26	.15
Easiness Reflection Time (RfT)	.19	-.30	.22	.07	.18	.10	-.11	.02	.24	-.14	-.17	**-.39***	.27	.06	.01	.06
**Happiness**																
Reaction Time (RT)	.25	-.22	.19	.08	.14	.14	-.25	.01	-.01	-.29	-.11	**-.35***	.24	.12	-.09	-.09
d’	-.06	**.37***	-.09	.04	-.09	-.11	.17	-.02	.02	.17	.05	.27	-.15	.04	.22	.09
c	**.01**	.07	-.27	-.04	.24	-.22	-.20	-.27	-.22	-.20	.29	.07	-.26	-.12	.17	.13
Emotion Labeling Reflection Time (RfT)	-.03	-.25	-.29	-.03	.12	-.18	-.33	-.06	.13	-.10	-.06	-.04	.24	-.01	-.17	.19
Easiness response	-.17	.07	-.14	-.13	-.23	-.23	-.10	.01	-.05	-.06	**-.35***	-.23	.12	-01	**-.35***	-.09
Easiness Reflection Time (RfT)	.28	-.30	.20	.07	.16	.05	-.06	-.04	.21	-.15	-.15	**-.35***	.19	.19	.01	.05
**Fear**																
Reaction Time (RT)	.27	-.13	.27	.03	-.04	.24	-.29	-06	-.04	**-.40***	-.18	-.32	.25	-.03	-.08	-.13
d’	-.13	.17	-.14	.13	-.03	-.29	.26	-.03	-.21	-.08	.19	.05	-.12	-.12	.06	-.11
c	-.22	**-.41***	-.12	-.10	-.29	-.14	-.15	-.14	-.17	-.22	-.22	-.08	.14	-.10	**-.39***	-.02
Emotion Labeling Reflection Time (RfT)	.13	-.04	-.29	.10	**.35**	-.11	-.23	.08	**.34***	.15	-.17	-.05	.24	.04	.09	.07

Easiness response	-.25	.01	.02	-.09	**-.34***	-.21	.06	-.01	-.13	.10	-.17	-.01	-.02	.13	-.28	.17
Easiness Reflection Time (RfT)	.18	**-.34***	.10	.13	.21	.14	-.14	-.04	.26	-.19	-.17	**-.37***	.27	-.01	-.03	.03
**Surprise**																
Reaction Time (RT)	.17	-.17	.22	-.04	.02	.16	**-.34***	.01	-.04	**-.34***	-.05	**-.34***	.24	.07	-.07	-.01
d'	-.14	.15	-.20	-.19	-.08	**-.34***	.02	-.28	-.23	-.19	.25	.23	.17	-.19	-.07	-.23
c	-.12	-.03	.25	-.17	-.08	.18	.01	.17	-.07	.03	-.09	-.11	-.20	.01	.04	-.01
Emotion Labeling Reflection Time (RfT)	-.17	-.07	-.29	.12	.15	-.10	-.22	.05	.30	.15	-.06	-.07	.27	.02	-.10	.31
Easiness response	-.06	.11	.13	-.07	-.25	-.14	-.03	-.01	-.11	-.04	.21	-.15	.15	.14	-.17	.02
Easiness Reflection Time (RfT)	.13	**-.43****	.10	.15	**.37***	.14	-.06	.06	.24	-.09	-.16	**-.34***	.25	.02	.04	-.01
**Sadness**																
Reaction Time (RT)	.20	-.13	.27	-.15	.04	.24	**-.40***	-.02	-.06	**-.36***	-.04	-.33	.24	.01	-.15	-.19
d'	-.16	.20	-.12	-.13	.09	-.31	.06	-.23	-.06	-.12	.13	.19	-.20	.01	.02	.02
c	.11	.15	-.12	**.43***	.25	-.02	.10	-.08	.27	.24	.04	.02	-.02	-.04	.11	.14
Emotion Labeling Reflection Time (RfT)	.21	-.01	.08	.11	.14	.03	-.31	.01	.27	.06	-.13	-.12	.27	.17	.07	.27
Easiness response	-.26	.15	-.17	-.06	-.30	-.23	.05	.08	-.01	.18	.02	.23	-.03	.16	-.20	.17
Easiness Reflection Time (RfT)	.22	-.28	.10	.15	.21	.05	-.17	-.10	.23	-.19	-.16	**-.38***	.25	.04	.04	.06

Note: FICSOs = Forensic Inpatients who have Committed Sexual Offenses; LoS = Length of Stay; Mood = Mood Disorders; SUD = Substance Use and Abuse Disorder: Psy = Psychotic Disorders: Anx = Anxiety Disorders; Avoi = Avoidant Personality Disorder; Depe = Dependent Personality Disord er; Ob-Co = Obsessional-Compulsive Personality Disorder; Pa-Ag= Passive-Aggressive Personality Disorder; Dep = Depressive Personality Disorder; Para = Paranoiac Personality Disorder; Schy = Schizotypal Personality Disorder; Schi = Schizoid Personality Disorder; Hist = Histrionic Personality Disorder; Narc = Narcissistic Personality Disorder; Bor = Borderline Personality Disorder; Anti = Antisocial Personality Disorder; $*p \leq .05$; $**p \leq .01$; $***p \leq .005$

Supplementary Table 3 – Non-parametric (Spearman's ρ) correlations between psychiatric diagnoses and facial expressions emotion recognition scores among the forensic inpatients who have committed non-sexual offenses (*n* = 25).

	FICNSOs (*n* = 25)																
	**Mood**	**SUD**	**Psy**	**Anx**	**Eat**	**Avoi**	**Depe**	**Ob-Co**	**Pa-Ag**	**Dep**	**Para**	**Schy**	**Schi**	**His**	**Narc**	**Bor**	**Anti**
**All emotions combined**																	
Reaction Time (RT)	.17	-.13	.39	-.05	N/A	.16	-.12	.02	-.20	-.29	-.22	**-.56****	N/A	-.14	-.24	-.18	.03
Mean Accuracy	-.15	.26	.03	.15	N/A	.07	.01	.03	-.06	.08	.15	.22	N/A	.31	.18	.27	-.19
Emotion Labeling Reflection Time (RfT)	-.18	-.03	-.12	.12	N/A	.27	-.03	.22	.01	.11	-.04	-.11	N/A	.23	.16	-.07	-.17
Easiness response	.10	-.06	-.04	-.03	N/A	.08	.10	.05	-.03	-.17	-.13	-.20	N/A	-.06	-.06	-.08	.13
Easiness Reflection Time (RfT)	-.39	.13	-.05	.01	N/A	.24	.12	.10	-.17	.01	.11	-.06	N/A	.01	-.12	.18	-.32
**Anger**																	
Reaction Time (RT)	.26	.01	.29	.15	N/A	.24	-.03	.20	.01	-.11	-.04	-.39	N/A	-.23	-.16	-.02	.04
d'	-.03	.13	.25	-.01	N/A	.01	-.03	-.04	-.08	.12	.17	.14	N/A	.28	.14	.21	-.18
c	.04	**-.43***	-.19	.25	N/A	-.05	-.04	-.06	-.03	-.36	**-.40***	-.35	N/A	.28	.18	**-.44***	-.02
Emotion Labeling Reflection Time (RfT)	-.12	.01	-.30	.05	N/A	.16	-.19	.17	.06	.12	-.01	-.17	N/A	.20	.18	.01	-.29
Easiness response	.05	.02	.06	-.04	N/A	.06	.25	.06	.01	-.10	-.16	-.04	N/A	-.06	-.03	-.07	.22
Easiness Reflection Time (RfT)	-.26	.09	-.06	.03	N/A	.27	-.05	.10	-.20	.09	.19	-.06	N/A	-.23	-.31	.23	-.29
**Disgust**																	
Reaction Time (RT)	.04	-.09	.30	-.10	N/A	.24	-.19	.02	-.31	-.27	-.29	**-.57****	N/A	-.17	-.35	-.06	-.01
d'	.03	-.14	.10	.26	N/A	.29	-.18	.16	-.13	-.16	-.13	-.20	N/A	.34	.15	-.13	-.12
c	.12	**.42***	.17	-.12	N/A	.12	.11	.16	.10	.26	.21	.20	N/A	-.25	-.11	**.49***	.23
Emotion Labeling Reflection Time (RfT)	-.10	.10	-.05	.02	N/A	.20	.07	.26	.14	.18	.21	-.04	N/A	-.23	-.06	.13	-.18
Easiness response	.10	-.12	-.06	.03	N/A	.03	.26	.08	.09	-.16	-.18	-.08	N/A	.18	.20	-.11	.10
Easiness Reflection Time (RfT)	-.36	.24	.03	-.10	N/A	.20	.08	.03	-.23	.01	.15	-.06	N/A	-.20	-.31	.25	-.22
**Happiness**																	
Reaction Time (RT)	.11	-.15	**.42***	-.15	.28	.22	-.14	.01	-.31	-.35	-.25	**-.54*****	N/A	.03	-.20	-.12	-.30
d'	.10	.01	-.14	.11	-.14	.11	-.14	-.01	-.17	.23	.18	.25	N/A	-.06	-.16	-.01	.35
c	.01	-.26	.01	.08	-.31	-.01	-.21	-.11	-.17	-.20	-.18	.01	N/A	.01	-.12	-.04	-.13
Emotion Labeling Reflection Time (RfT)	-.28	-.02	.01	-.02	.20	.18	.19	.10	-.08	-.01	-.14	.01	N/A	.11	.02	-.02	-.33
Easiness response	-.03	.24	-.18	-.07	-.14	.25	.09	.21	.01	.11	.18	-.08	N/A	-.11	-.08	.11	.13
Easiness Reflection Time (RfT)	-.39	-.14	-.02	.03	.20	.33	.05	.12	-.25	-.09	-.11	-.14	N/A	.28	.02	-.06	-.30
**Fear**																	
Reaction Time (RT)	.07	-.01	**.42***	-.14	.28	.18	-.12	.01	-.25	-.29	-.21	**-.56*****	N/A	-.11	-.27	-.09	-.04
d'	-.07	**.43***	.25	-.12	.34	-.02	.24	.07	.14	.12	.15	.15	N/A	.31	.33	.33	-.37
c	-.10	-.07	.01	.07	-.25	.35	.19	.27	-.03	.03	-.08	.01	N/A	-.34	-.27	-.11	-.06
Emotion Labeling Reflection Time (RfT)	-.22	.17	-.16	.05	.34	.14	-.05	.17	.08	.21	.08	.03	N/A	.23	.22	.17	-.04

Easiness response	.15	-.05	-.06	-.04	-.28	-.12	.18	-.09	.01	-.29	-.19	-.20	N/A	-.03	-.01	-.05	.14
Easiness Reflection Time (RfT)	**-.54*** **	.27	-.15	.03	.28	.24	.15	.10	-.17	-.01	.10	-.04	N/A	-.06	-.16	.19	-.35
**Surprise**																	
Reaction Time (RT)	.11	-.19	.36	-.08	.28	.16	-.20	.01	-.23	-.30	-.29	**-.56** **	N/A	-.06	-.20	-.27	.10
d'	.17	-.21	.08	.26	-.03	.10	.01	.05	-.06	.04	.08	.26	N/A	.01	-.04	.02	.01
c	-.32	.18	-.27	-.36	.17	-.14	-.02	-.15	-.06	.03	.08	-.14	N/A	.03	-.02	.06	-.14
Emotion Labeling Reflection Time (RfT)	-.03	-.08	.13	-.26	.31	.08	-.19	.01	-.11	.09	-.05	-.18	N/A	.28	.12	.01	-.19
Easiness response	.08	-.05	-.09	-.07	-.28	.06	.12	.01	-.07	-.15	.01	-.18	N/A	-.11	-.13	-.07	.08
Easiness Reflection Time (RfT)	**-.46***	-.01	-.05	-.02	.31	.22	.22	.15	-.06	.12	.15	.06	N/A	.20	.10	-.11	**-.44***
**Sadness**																	
Reaction Time (RT)	.29	-.09	.38	.05	.17	.12	.08	.12	.03	-.18	-.11	-.39	N/A	-.11	-.06	-.17	.15
d'	-.21	.14	.06	.16	.23	.18	.03	.21	.10	.17	.08	.21	N/A	.28	.28	.06	-.20
c	.12	-.35	.01	-.06	-.23	**-.43***	-.23	-.30	.10	-.32	-.33	-.30	N/A	.28	.28	**-.44***	-.03
Emotion Labeling Reflection Time (RfT)	-.07	-.25	.10	.02	.11	.20	-.34	-.02	-.31	-.21	-.24	-.35	N/A	.34	.02	-.23	-.15
Easiness response	.13	-.06	-.06	-.14	-.20	.04	-.01	-.02	-.08	-.17	-.14	-.33	N/A	-.06	-.10	-.02	-.01
Easiness Reflection Time (RfT)	**-.40***	.14	-.20	.19	.28	.31	.26	.26	.01	.09	.15	.01	N/A	.06	.04	.11	-.34

Note: FICNSOs = Forensic Inpatients who have Committed Non-Sexual Offenses; LoS = Length of Stay; Mood = Mood Disorders; SUD = Substance Use and Abuse Disorder: Psy = Psychotic Disorders: Anx = Anxiety Disorders; Eat = Eating Disorders; Avoi = Avoidant Personality Disorder; Depe = Dependent Personality Disorder; Ob-Co = Obsessional-Compulsive Personality Disorder; Pa-Ag= Passive-Aggressive Personality Disorder; Dep = Depressive Personality Disorder; Para = Paranoiac Personality Disorder; Schy = Schizotypal Personality Disorder; Schi = Schizoid Personality Disorder; Hist = Histrionic Personality Disorder; Narc = Narcissistic Personality Disorder; Bor = Borderline Personality Disorder; Anti = Antisocial Personality Disorder; $*p \leq .05$; $**p \leq .01$; $***p \leq .005$

Supplementary Table 4 – Non-parametric (Spearman's ρ) correlations between medication and scores of facial expressions recognition among the forensic groups.

	**FICSOs (n = 37)**							**FICNSOs (n = 25)**						
	**Anxiolytic**	**Typ. Antipsychotic**	**Atyp. Antipsychotic**	**Antidepressant**	**Anticonvulsant**	**Hormone**	**Other**	**Anxiolytic**	**Typ. Antipsychotic**	**Atyp. Antipsychotic**	**Antidepressant**	**Anticonvulsant**	**Horm**	**Other**
**All emotions combined**														
Reaction Time (RT)	.11	.22	.23	.17	.29	.05	.26	.05	.37	.10	-.01	.07	.08	**-.45***
Mean Accuracy	.19	.23	-.04	-.07	.17	-.07	.11	-.37	-.32	.28	.35	.08	.17	-.18
Emotion Labeling Reflection Time (RfT)	.07	-.09	.09	.17	.08	.02	.24	.01	.09	.12	.16	-.01	-.28	-.27
Easiness response	.13	.06	-.30	-.01	.13	.02	.28	.20	.18	-.16	-.30	-.01	.06	-.18
Easiness Reflection Time (RfT)	.03	-.04	.16	**.35***	.06	.03	.07	.11	.19	.39	**.45***	**.43***	.23	-.13
**Anger**														
Reaction Time (RT)	.04	.16	.30	.22	.30	.09	.18	-.02	.28	.22	.12	.06	.11	**-.60*****
d'	.15	.13	-.03	-.06	.09	-.18	.18	**-.49***	-.27	**.41***	.19	-.02	.11	-.18
c	-.09	.03	.07	-.03	.11	.01	-.29	.12	-.12	.04	.25	-.04	-.14	-.03
Emotion Labeling Reflection Time (RfT)	-.05	.03	.20	.26	.22	-.02	.14	.01	.04	-.02	.01	-.16	-.25	-.20
Easiness response	.12	.05	-.30	-.01	.11	.01	**-.33***	.20	.22	-.02	-.21	.08	-.14	.01
Easiness Reflection Time (RfT)	.01	.01	.17	**.33***	.10	-.01	-.06	.07	.13	.33	.39	.37	.23	-.27
**Disgust**														
Reaction Time (RT)	-.02	.23	.20	.18	.14	-.02	.20	-.06	.25	.12	-.12	-.02	-.08	**-.44***
d'	.22	.25	-.10	-.14	.18	-.06	-.05	-.29	-.37	.12	.25	-.12	.03	-.22
c	-.09	-.05	-.11	.07	-.26	.11	.19	-.07	.06	.12	-.30	.21	-.06	-.02
Emotion Labeling Reflection Time (RfT)	.04	.05	.06	.10	.17	-.04	.21	.15	.25	.12	.09	.16	.08	-.36
Easiness response	.09	.02	-.30	.02	.16	.01	.29	.06	.04	-.08	-.28	-.13	-.07	-.02
Easiness Reflection Time (RfT)	.07	.03	.15	**.34***	.09	.01	.07	.22	.30	**.43***	**.44***	**.58****	.31	-.09
**Happiness**														
Reaction Time (RT)	.06	.15	.23	.25	.23	.04	.21	-.09	.02	.02	.16	.16	.14	**-.59****
d'	.15	.14	-.06	.16	.03	-.03	**.35***	-.12	.04	-.10	-.29	-.31	-.06	-.07
c	-.04	.17	.12	.06	.07	.02	.03	-.17	**-.54****	.06	-.06	-.18	.01	-.07
Emotion Labeling Reflection Time (RfT)	.01	-.05	.12	.11	-.06	-.02	.27	.01	-.09	.14	**.43***	.29	-.25	-.16
Easiness response	.15	-.09	-.24	-.10	.17	.13	.24	.14	.24	.01	-.12	.18	.17	-.19
Easiness Reflection Time (RfT)	-.03	.02	.25	**.39***	.10	.11	.13	.01	-.01	.27	**.42***	.25	-.06	-.06
**Fear**														
Reaction Time (RT)	.11	.15	.22	.15	.32	.12	.21	-.01	.36	.12	.04	.14	.11	-.39
d'	-.04	.09	-.11	-.31	-.08	-.20	.01	**-.46***	-.26	.35	.23	.08	.07	-.04
c	-.02	-.08	-.18	.09	-.07	.29	-.13	.01	.14	-.02	**.47***	.07	-.21	.23

Emotion Labeling Reflection Time (RfT)	-.04	-.18	.06	.06	-.04	.09	.11	.15	.26	.10	.09	.12	-.23	.02
Easiness response	.15	.19	-.27	-.02	.18	.04	.21	.21	.20	-.23	-.30	.01	.16	-.16
Easiness Reflection Time (RfT)	-.04	-.07	.11	**.35***	.03	.01	.09	.12	.20	.35	**.43***	.33	.23	-.04
**Surprise**														
Reaction Time (RT)	.27	.30	.14	.21	**.34***	-.05	**.38***	.15	**.43***	-.10	-.05	-.01	-.03	-.28
d’	.07	.26	-.08	-.05	.29	.14	-.01	-.26	-.36	-.04	.01	-.18	.08	-.25
c	-.08	-.11	.08	-.01	-.18	-.16	.17	.22	**.42***	-.08	-.08	.16	.11	.22
Emotion Labeling Reflection Time (RfT)	.11	-.10	-.03	.06	-.06	.16	.15	-.09	.17	.02	.06	.03	-.28	-.08
Easiness response	.30	.15	-.21	.03	**.39***	-.01	.24	.16	.16	-.22	-.36	.02	.28	-.29
Easiness Reflection Time (RfT)	.02	-.13	.08	.29	.07	-.03	.01	-.04	.11	.20	**.42***	.20	.08	-.15
**Sadness**														
Reaction Time (RT)	.21	**.35***	.28	.13	**.41***	.08	.27	.12	**.46***	.10	.03	.08	.11	-.37
d’	.06	.16	-.12	-.02	-.14	-.20	.07	-.23	-.26	-.06	**.45***	.02	-.16	.07
c	-.19	-.01	.05	-.12	-.08	-.15	-.19	.11	-.11	-.33	-.29	-.35	-.07	.32
Emotion Labeling Reflection Time (RfT)	.11	-.08	.18	.16	.15	.11	.19	-.25	-.09	.08	-.01	-.25	-.08	-.33
Easiness response	.21	.19	-.18	-.10	.06	.10	.27	.06	.14	-.23	-.31	-.08	.03	-.27
Easiness Reflection Time (RfT)	.08	-.11	.12	**.37***	.06	.06	.10	.04	.09	**.45***	.39	.25	.14	-.17

Note: FICSOs = Forensic Inpatients who have Committed Sexual Offenses; FICNSOs = Forensic Inpatients who have Committed Non-Sexual Offenses; Typ. Antipsychotic = Typical Antipsychotic; Atyp. Antipsychotic = Atypical Antipsychotic; $*p \leq .05$*;* $**p \leq .01$*;* $***p \leq .005$
